# The Effect of Safflower Yellow on Spinal Cord Ischemia Reperfusion Injury in Rabbits

**DOI:** 10.1155/2013/692302

**Published:** 2013-12-07

**Authors:** Daiwei Zhou, Bingbing Liu, Xiaoshan Xiao, Peng Dai, Songmei Ma, Weihua Huang

**Affiliations:** ^1^Department of Anesthesiology, Guangdong No. 2 Provincial People's Hospital, Guangdong Provincial Emergency Hospital, Guangzhou, Guangdong Province 0086 510317, China; ^2^Department of Anesthesiology, Foshan First People's Hospital, Guangdong Province 0086 510317, China

## Abstract

Safflower yellow (SY) is the safflower extract and is the one of traditional Chinese medicine. The aim of the present work was to investigate the effect of SY on spinal cord ischemia reperfusion injury (SCIRI) in rabbits. The models of spinal cord ischemia reperfusion (SI/R) were constructed, and the degree of the post-ischemic injury was assessed by means of the neurological deficit scores and plasma levels of lipid peroxidation reactioin and neuronal morphologic changes. SCIRI remarkably affected the functional activities of the hind limbs and activated lipid peroxidation reaction. SY could attenuate apoptosis and SCIRI by enhancing Bcl-2 expression and inhibiting Bax and caspase-3 activation.

## 1. Introduction

According to the pathophysiologic features, spinal cord injury is mainly categorized into primary injury and secondary injury. Primary injury mainly includes direct injury and ischemic injury, and it often occurs in a relatively short period of time after injury (generally considered as early as 4 h after injury), with the irreversible nerve damage [1]. The perfusion after spinal cord ischemia may further aggravate the damage and cause spinal cord ischemia reperfusion injury (SCIRI). SCIRI is one of the most frequent types of secondary spinal cord injury and it aggravates the neuro functional impairment of the limbs. Secondary injury generally lasts longer time, up to 7 days or longer, and the secondary nerve damage can be reversed by means of appropriate interventions [2]. Spinal cord ischemia reperfusion injury (SCIRI) is defined as follows: after removing the factors that cause spinal cord ischemia and the recovery of spinal cord blood supply, its neuronal function cannot be improved and its ischemia injury is more intense than its original level, or even present in the irreversible tardive dead phenomenon on spinal cord neurons [3]. It was generally considered that the reasons for SCIRI included oxygen free radical-induced lipid peroxidation, leukocyte activation, and inflammatory and neuronal apoptosis. In recent years, although there have been many clinical treatments of spinal cord injury, the results were not satisfactory.

Safflower yellow (SY) is extracted from the flowers of the plant safflower (*Carthamus tinctorius*) and as the traditional Chinese medicine it has been extensively used for the treatment of cardio cerebrovascular diseases. SY can promote blood circulation, remove blood stasis, and thereby improve capillary circulation at the site of tissue injury [2]. SY is mixtures of a water-soluble chalcone component, in which both hydroxyl safflower yellow A (HSYA) and safflower yellow B (SYB) are the main components. It has been shown that safflower injection excellently protected the heart by way of improving functions of cardiac contraction and dilation, increasing coronary blood flow, and strengthening the bcl-2 (anti apoptosis gene) protein expression [4]. Additionally, our previous study indicated that SY alleviated the injured tendon adhesion and inflammatory reaction and promoted the repair of injured tendon [2]. However, it is still unknown whether SY can effectively protect against SCIRI. Therefore, this study constructed SCIRI models with New Zealand rabbits to determine the degree of spinal cord injury and the protective effect of SY on SCIRI.

## 2. Materials and Methods

### 2.1. Animals

The adult male New Zealand rabbits (body weight 2.0–2.5 kg), obtained from the Animal Experimental Center of Southern Medical University, were used in this study. All procedures are in strict accordance with the protocols approved by the Chinese institutional ethics committee. The rabbits were housed in individual cages in a temperature-controlled room (22–25°C) and acclimated for 1 week before experiments. Food was removed 8 h prior to the study, but all animals had free access to water.

### 2.2. Model Establishment

After being anesthetized with ketamine (10 mg/kg body weight, intramuscularly), the airway was maintained by endotracheal intubation (ID = 3.5 mm, depth 10–12 cm) and mechanical ventilation was performed with a 35–45 mmHg P_ET_CO2. The polyethylene catheter was inserted into the right femoral artery for monitoring the mean arterial pressure (MAP) and drawing blood samples, and the one was did into the right femoral vein for solution infusion. The abdominal aorta was exteriorized by midline laparotomy. The SCIRI model was established by occluding the abdominal aorta under the left renal artery for 40 min (when the abdominal aorta was occluded MAP decreased to 0 mmHg) followed by reperfusion as described [5].

### 2.3. Experimental Protocol

Twenty-four rabbits were randomly divided into three groups (*n* = 6 per group): sham-operated control (Cont), spinal cord ischemia reperfusion (SI/R), and SI/R treated with safflower yellow (SI/R + SY). The control group only executed anesthesia and surgical procedures, except for occluding the abdominal aorta. The SI/R + SY group was intravenously injected with 2 mL/kg of a solution of 16% (wt/vol) SY (1 mL, containing 1.6 mg SY, Z20050146; Yongning Pharma, Zhejiang Province, China), followed by continuous infusion of a total of 5 mL/kg through the right femoral vein at the moment of reperfusion beginning after 40 minutes of the abdominal aorta occlusion. The same volumes of 0.9% saline solution were administrated in control and SI/R groups. Blood samples were obtained at the end of 0 hour (*T*0), 4 hours (*T*1), 12 hours (*T*2), 24 hours (*T*3), and 48 hours (*T*4) after reperfusion, and the plasma was separated and stored at −80°C for further analysis. All animals were sacrificed 48 hours after reperfusion and were rapidly perfused with 0.9% sodium chloride, and the L2-5 segments of the spinal cord were quickly removed. The L2-3 segment in each animal was used for western blot, and the other segment (L4-5) was immersed into 10% neutral formaldehyde for 2-3 days and was used for morphology analysis.

### 2.4. Neurological Deficit Scores for Hind Limbs

At the end of 4 hours (h), 12 h, 24 h, and 48 h after reperfusion, neurological deficit scores of hind limbs were recorded according to the criterion as follows [6].

 0: hind limbs are absolutely paralysed and cannot move.
 1: hind limbs move slightly but cannot move against gravity.
 2: hind limbs can move but cannot walk or jump.
 3: hind limbs can walk and jump with obvious ataxia.
 4: hind limbs can jump normally.

### 2.5. Measurement of MDA, SOD, and IL-8 with ELISA

Plasma levels of MDA, SOD, and IL-8 were determined by using the commercially available rabbit ELISA kit (BenderMed, Vienna, Austria). Plasma samples (in PBS) were purified using Affinity Sorbent and Affinity Column (Cayman chemical, Ann Arbor, MI) and then processed for analysis, according to the instructions provided by the manufacturer (Quantikine; R&D, Minneapolis, MN). The optical density (OD) value was measured at 490 nm wavelength. The OD values of the blank control were subtracted from those of every standard sample, and then the standard curves were drafted. The levels of MDA, SOD, and IL-8 were calculated according to the standard curve as the methods described in our prior study [2].

### 2.6. Western Blot Assay for Caspase-3

Frozen spinal cord tissues were homogenized using lysis buffer and then were centrifuged at 15000 g for 40 min at 4°C, and protein concentrations were determined using the Bradford assay (Bio-Rad, USA). 50 *μ*g total protein was separated by sodium dodecyl sulfate-polyacrylamide gel electrophoresis (10% SDS-PAGE) and transferred to PVDF membranes (Millipore). Membranes were incubated in blocking buffer (5% skim milk in TBST), then with rabbit anticaspase-3 (1 : 800, Abcam, USA), and GAPDH (1 : 2000, Cell Signaling Technology, Beverly, MA) in TBST overnight at 4°C [7]. Incubated membranes were then treated with secondary antibody conjugated with horseradish peroxidase in TBST for 2 h at 37°C. Blots were developed by enhanced chemiluminescence and digitally scanned. The optical density of each resulting labeled band was measured in an image analysis program.

### 2.7. HE Staining

After immersing in 10% neutral formaldehyde for 2-3 d, the spinal cord samples (L4-5 segments) were fixed and dehydrated through a graded ethanol series, embedded, and sectioned at 5 mm on a frozen microtome and then mounted and covered. The sections were routinely dewaxed and hydrated, and as our previous study described [2] they were stained by HE and dehydrated, cleared, and covered.

According to neuronal morphological criteria [8], the anterior horn neurons were observed at the magnification of 200 times. Five high-powered visual fields were randomly selected on every section and eight sections were randomly selected in each animal. The anterior horn motor neurons were counted and the normal neurons to total neurons ratios were calculated.

### 2.8. TUNEL Immunohistochemical Staining

TUNEL staining was used to detect the expression of apoptosis protein in the spinal cord anterior horn neurons in rabbits. The sections were stained with TUNEL (In Situ Cell Death Detection Kit, POD; Roche, Basel) according to the manufacturer's instructions. Five dark visual fields were randomly selected on every section, and the TUNEL-positive neurons and the total numbers of neurons in the selective visual fields were counted. TUNEL-positive index (the TUNEL-positive to whole neurons ratio) was calculated. Eight sections of each animal from all groups were used for measurement, and five high-powered visuals from every section were randomly selected to measure the TUNEL-positive indexes.

### 2.9. Immunohistochemical Staining for Bax and Bcl-2

To detect the expression of proapoptotic protein and antiapoptotic protein, the spinal cord sections were, respectively, stained with Bax and Bcl-2 (Santa Cruz, CA, USA) according to the manufacturer's instructions. Five high-powered visual fields were randomly selected on every section, and eight sections were selected in each animal. OD values of Bax and Bcl-2 positive neurons were, respectively, measured with Image-Pro plus 6.0 software. The OD value was the sum of all positive neurons pixel OD values divided by the areas of the spinal cord anterior horn regions.

*Statistical Analysis*. All experimental data were expressed as means ± SD. The statistical significance of the results was evaluated by one-way ANOVA with SPSS 17.0 software, and *P* < 0.05 was considered significant.

## 3. Results

### 3.1. Basic Data

No animal died during the experimental period. Five cases of postoperative urinary retention were improved after bladder massage. Basic vital signs of all animals including heart rate (HR) and MAP were stable and there were no significant differences between groups (Table 1). Postoperative abdominal incisions grew well without flare and purulent secretion.

### 3.2. Neurological Deficit Scores of Hind Limbs

**Figure 1:**
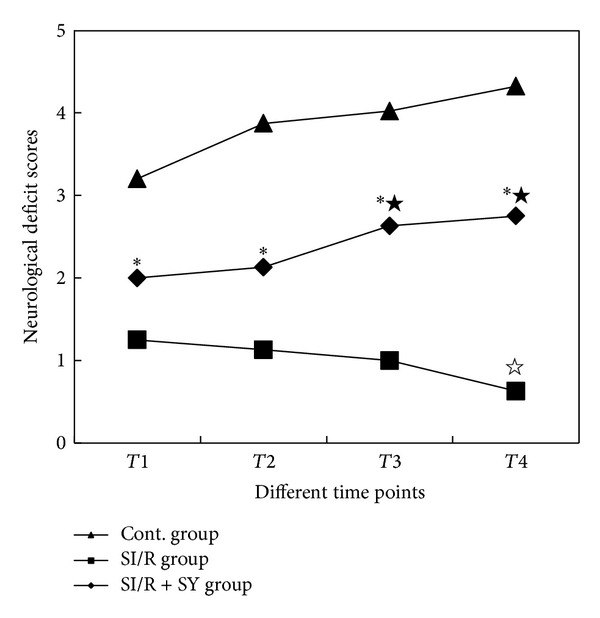
Neurological deficit scores of hind limbs at the different time points. ∗ indicates comparison with control (Cont) group and SI/R group in SI/R + SY group at the same time point, *P* < 0.0; ★ indicates comparison with *T*1 and *T*2 at *T*3 or *T*4 in SI/R + SY group, *P* < 0.05; *☆* indicates comparison with *T*1–*T*3 at *T*4 in SI/R group, *P* < 0.05. *T*1: 4 h after reperfusion; *T*2: 12 h after reperfusion; *T*3: 24 h after reperfusion; *T*4: 48 h after reperfusion.

Animal hind limbs appeared at different degrees of functional activity limitation in SI/R and SI/R + SY groups. Statistical results showed that the scores of SI/R + SY group were all higher than those of SI/R group at *T*1, *T*2, *T*3, and *T*4 time points (*P* < 0.05, Figure 1). In SI/R group, the scores were obviously lower at *T*4 than those at *T*1, *T*2, and *T*3 (*P* < 0.05). Animal functional activities of SI/R + SY group were better at *T*3 and *T*4 than at *T*1 and *T*2, but the comparison between *T*1 and *T*2 had no statistical significance (*P* > 0.05), and also no significant difference was seen between *T*3 and *T*4 (*P* > 0.05).

### 3.3. Levels of MDA and the Activities of SOD

**Figure 2:**
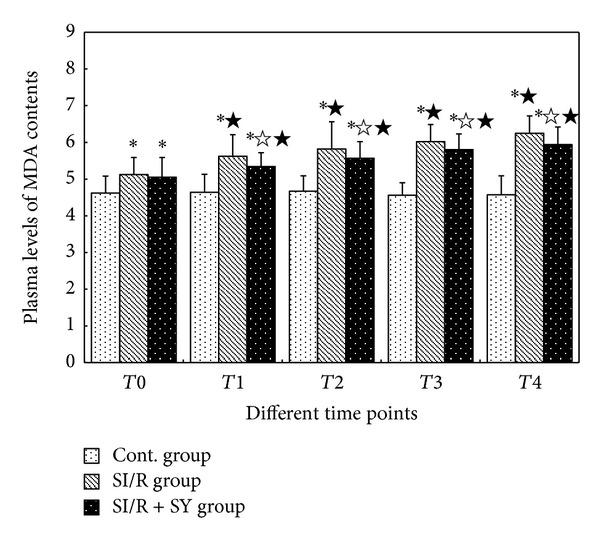
Changes of MDA levels at the different time points. ∗ indicates comparison with control (Cont) group at the same time point, *P* < 0.05; *☆* indicates comparison with SI/R group at the same time point, *P* < 0.05; ★ indicates comparison with *T*0 in the same group, *P* < 0.05. *T*0: 0 h after reperfusion; *T*1: 4 h after reperfusion; *T*2: 12 h after reperfusion; *T*3: 24 h after reperfusion; *T*4: 48 h after reperfusion.

The result showed that MDA levels at the different time points in the Control group were not obviously changed (*P* > 0.05, Figure 2). Plasma levels of MDA in the SI/R group were gradually increased from *T*1 to *T*4 (*P* < 0.05), and similar trend of progressive increase in MDA was also seen in the SI/R + SY group (*P* < 0.05). At *T*0 time point, the levels of MDA between SI/R and SI/R + SY groups did not show statistical significance (*P* > 0.05), but they were higher than those in the control group (*P* < 0.05). Beyond the *T*0 time point, at the same time point from *T*1 to *T*4, MDA levels were markedly lower in SI/R + SY group than in SI/R group (*P* < 0.05).

**Figure 3:**
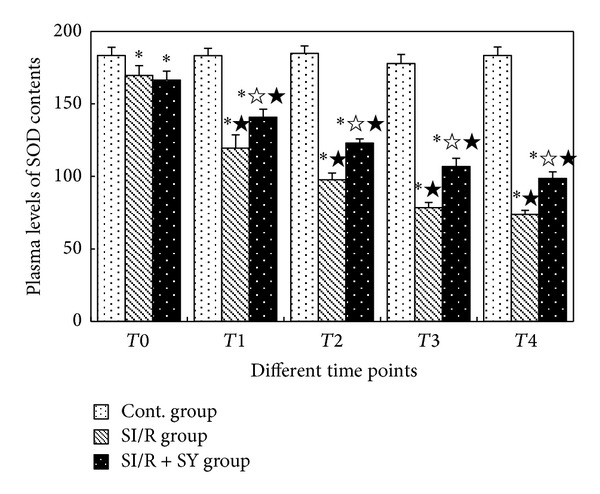
Changes of SOD activities at the different time points. *T*1: 4 h after reperfusion; *T*2: 12 h after reperfusion; *T*3: 24 h after reperfusion; *T*4: 48 h after reperfusion. ∗ indicates comparison with Cont. group at the same time point, *P* < 0.05; *☆* indicates comparison with SI/R group at the same time point, *P* < 0.05; ★ indicates comparison with *T*0 in the same group, *P* < 0.05.

Changes of SOD activities in Cont. group had no statistical significance at the different points (*P* > 0.05, Figure 3). At *T*0 time point, SOD activities of SI/R group and SI/R + SY group were markedly lower than those of Control group (*P* < 0.05), but the comparison between SI/R group and SI/R + SY group has no statistical significance (*P* > 0.05). From *T*1 to *T*4, SOD activities of the SI/R group decreased gradually, and they were all lower than those at *T*0 (*P* < 0.05); additionally, they were all lower than those at the same time points of Control group (*P* < 0.05). Compared with SI/R group, SOD activities of SI/R + SY group were higher at the same time point (*P* < 0.05), but they were all lower than those of the control group (*P* < 0.05).

### 3.4. Changes of the Serum Levels of IL-8

**Figure 4:**
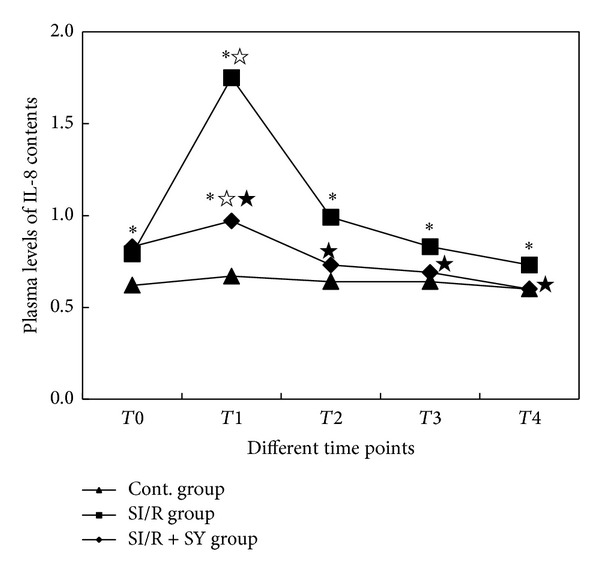
Changes of plasma IL-8 levels at the different time points. *T*1: 4 h after reperfusion; *T*2: 12 h after reperfusion; *T*3: 24 h after reperfusion; *T*4: 48 h after reperfusion; Cont, sham control. ∗ indicates comparison with the sham control group at the same time point, *P* < 0.05;☆ indicates comparison with SI/R group at the same time point, *P* < 0.05; ★ indicates comparison with *T*0 in the same group, *P* < 0.05.

Similar to the changes of MDA and SOD in the Control group, the expression levels of IL-8 did not show significant difference over time (*P* > 0.05, Figure 4), but it was higher in the SI/R group (*P* < 0.05 versus control) and was significantly reduced in the SI/R + SY group.

### 3.5. Caspase-3 Protein Expression

**Figure 5:**
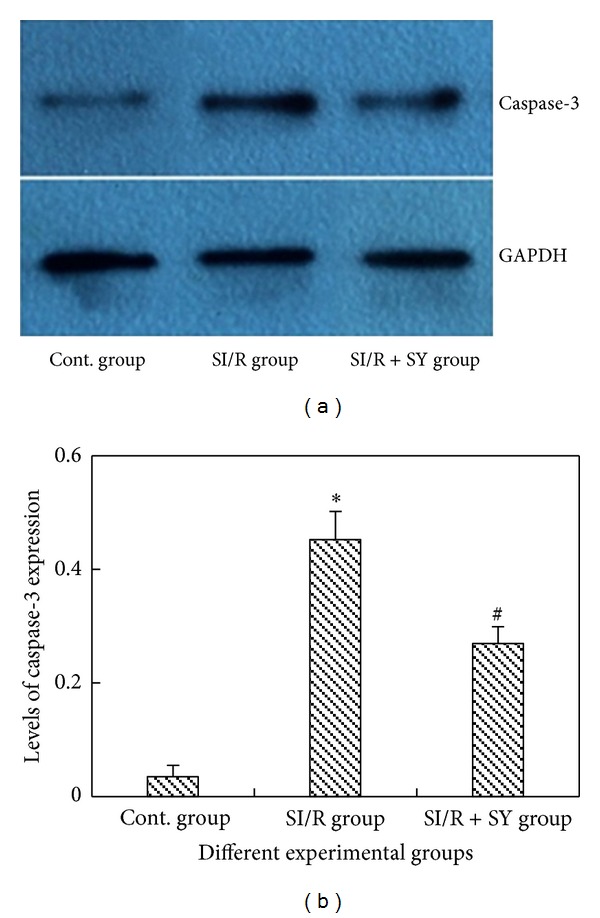
Western blot assay of caspase-3 apoptosis protein in three groups (a). Cont: sham control. Levels of caspase-3 expression were measured (b), and ∗ indicates comparison with the other two groups, *P* < 0.05; # indicates comparison with SI/R group, *P* < 0.05.

Western blotting (Figure 5) revealed that caspase-3 protein expression in the SI/R group was significantly increased as compared to the control group (*P* < 0.05), which was significantly attenuated in the SI/R + SY group (*P* < 0.05 versus SI/R, Figure 5(b)).

### 3.6. Morphological Changes of the Anterior Horn Neurons

**Figure 6:**
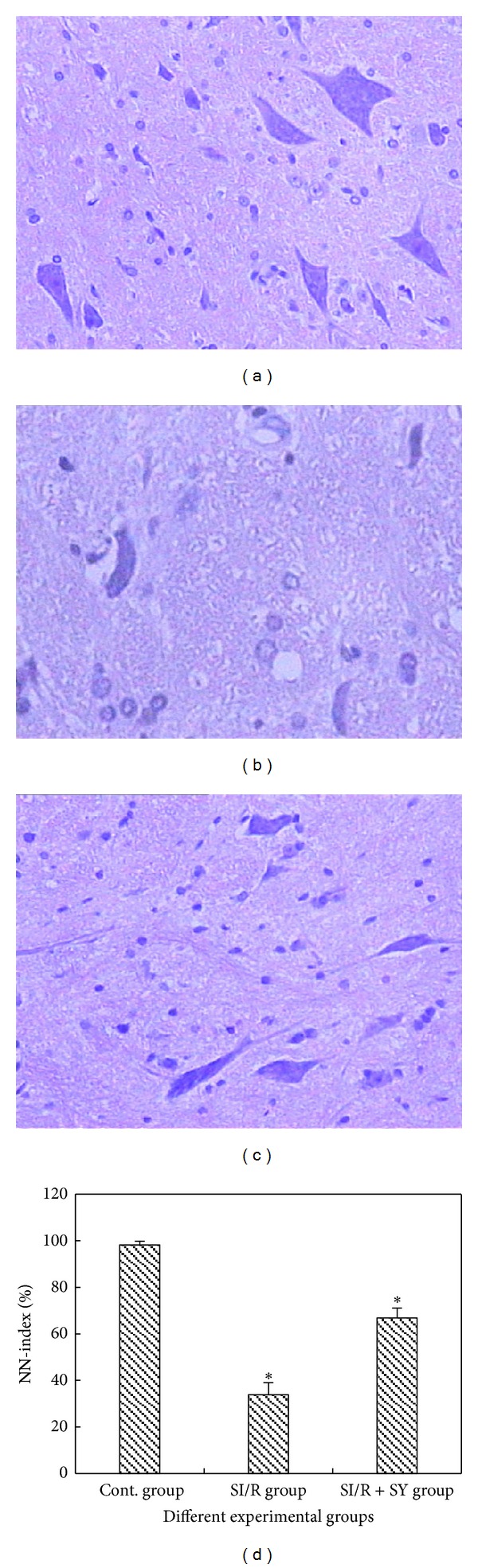
The neurons of spinal cord anterior horn were assessed by HE staining and viewed at the magnification of 200 times. There were numerous normal neurons without morphological change in the control (Cont.) group (a). Neuronal structural changes appeared in SI/R group (b), such as neuronal pyknosis, light staining tigroid body, and nucleus atrophy. The percentage of normal neurons was higher in the SI/R + SY group (c) as compared to that in the SI/R group, and only slight edema was observed in SI/R + SY group. Bar graph (d) showed the comparison for the ratio of normal motoneurons in spinal cord anterior horn between SI/R group and SI/R + SY group, and ∗ indicated comparison with the other groups, *P* < 0.05. NN-index: normal to total neuron number ratio.

*Histopathological Changes.* The sections with HE staining were observed at the light microscopic level by an investigator who was initially blinded in terms of group assignment. The results showed that the spinal cord motoneurons in the sham control group (Figure 6(a)) were morphologically normal with clear profile, polygonal perikaryon, and round nucleus. There were no vacuoles present surrounding these neurons. By contrast, in the SI/R group, the number of normal motoneurons was apparently reduced (Figure 6(b)). In addition, neuronal structural changes were observed, which included neuronal pyknosis, light staining tigroid body, nucleus atrophy and nucleolus disappearance, and so on. Furthermore, hemorrhagic macules were scattered into tissue structures and vacuolar changes were observed in the cytoplasm. Morphologic structures of neurons in SI/R + SY group were basically normal, except for slight edema (Figure 6(c)). Statistical analysis showed that the percentage of normal motoneurons was larger in the SI/R + SY group (66.75% ± 4.37% of all neurons) than that in the SI/R group (33.74% ± 5.31% of all neurons, *P* < 0.05; Figure 6(d)).

**Figure 7:**
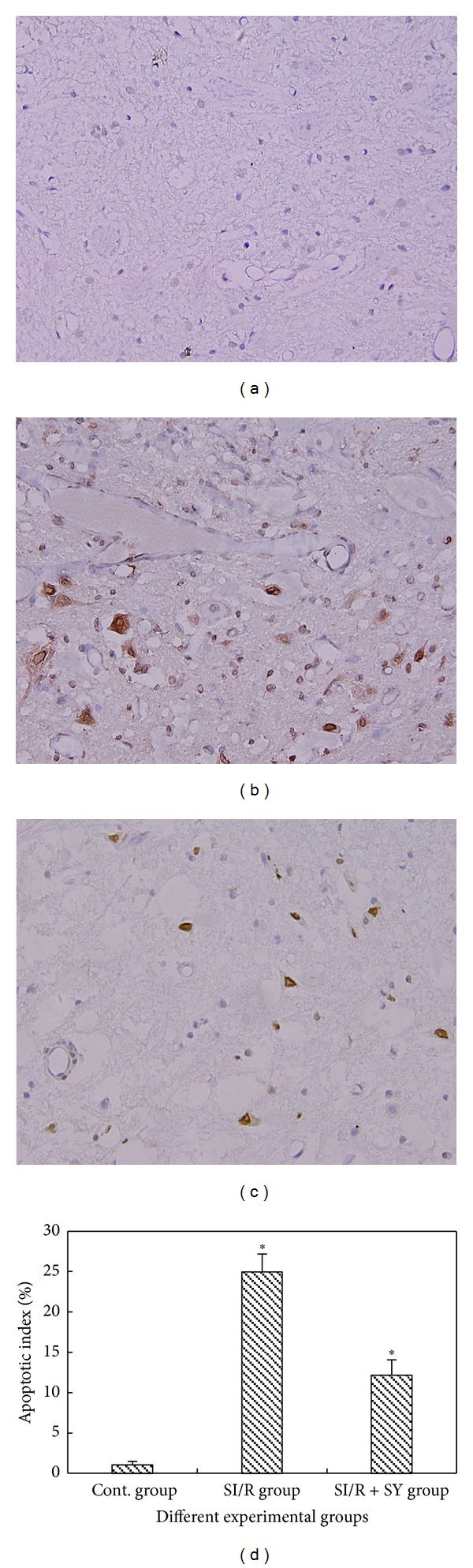
((a)–(d)) TUNEL immunohistochemical staining in the spinal cord anterior horn at the same magnification of 200 times. TUNEL-positive neurons were observed in SI/R group (b) and SI/R + SY group (c), but in the control group they were almost not detected (a). (d) The apoptotic index (TUNEL-positive to total neuron ratio) of anterior horn neurons, and ∗ indicates comparison with the two other groups, *P* < 0.05.

*Light Microscopic (LM) Observation of Apoptotic TUNEL-Positive Cells.* Spinal cord sections were stained with TUNEL and were observed at the high light microscopic level (200 times magnification, Figure 7). The results showed that neuronal structures in spinal cord anterior horn in the sham control group were basically normal with rare detectable TUNEL-positive staining (Figure 7(a)). In the spinal cord anterior horn of the SI/R group, amount of vacuoles appeared and a large number of TUNEL-positive neurons were observed (Figure 7(b)). By contrary, apoptosis neurons marked by TUNEL in the SI/R + SY group were obviously decreased (Figure 7(c)). Apoptotic indexes were calculated (the ratios of TUNEL-positive neurons to whole neurons) which showed that the apoptotic index was smaller in the SI/R + SY group than that in the SI/R group (*P* < 0.05, Figure 7(d)), despite the fact that the apoptotic indexes in the two injury groups were all higher than that in the control group.

**Figure 8:**
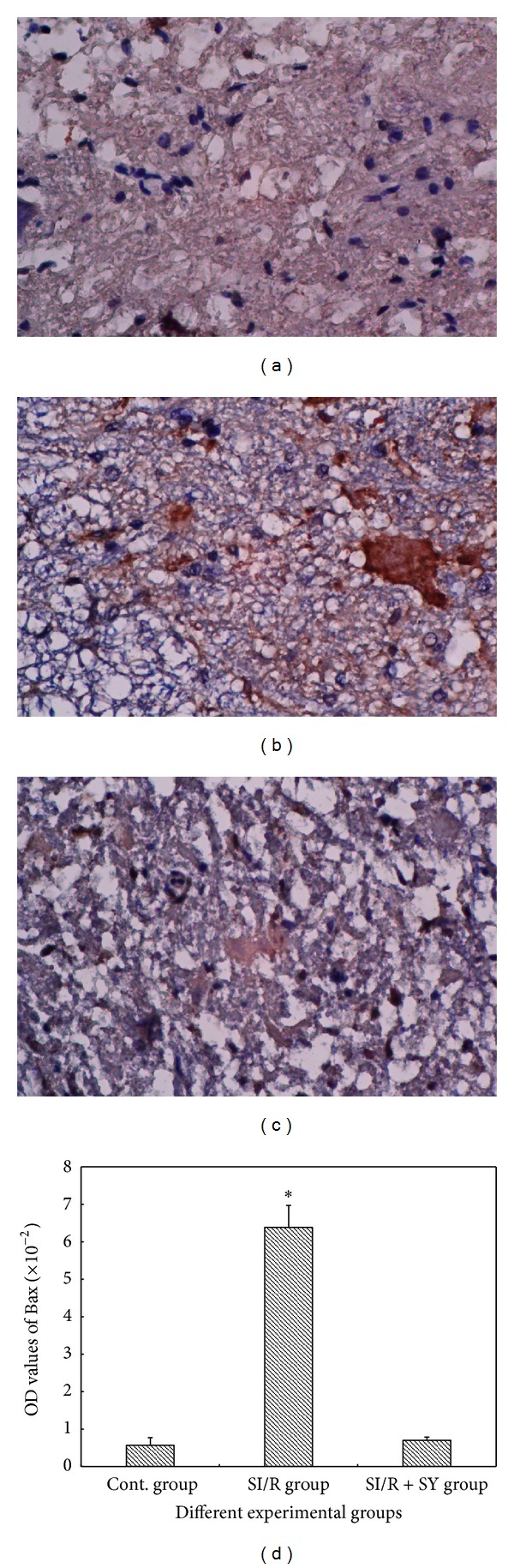
Images (a)–(c) showed Bax protein expression in the spinal cord anterior horn at the same magnification of 200 times, and OD values of Bax protein staining were measured and analyzed in image (d); ∗ indicate comparison with the two other groups, *P* < 0.05.

**Figure 9:**
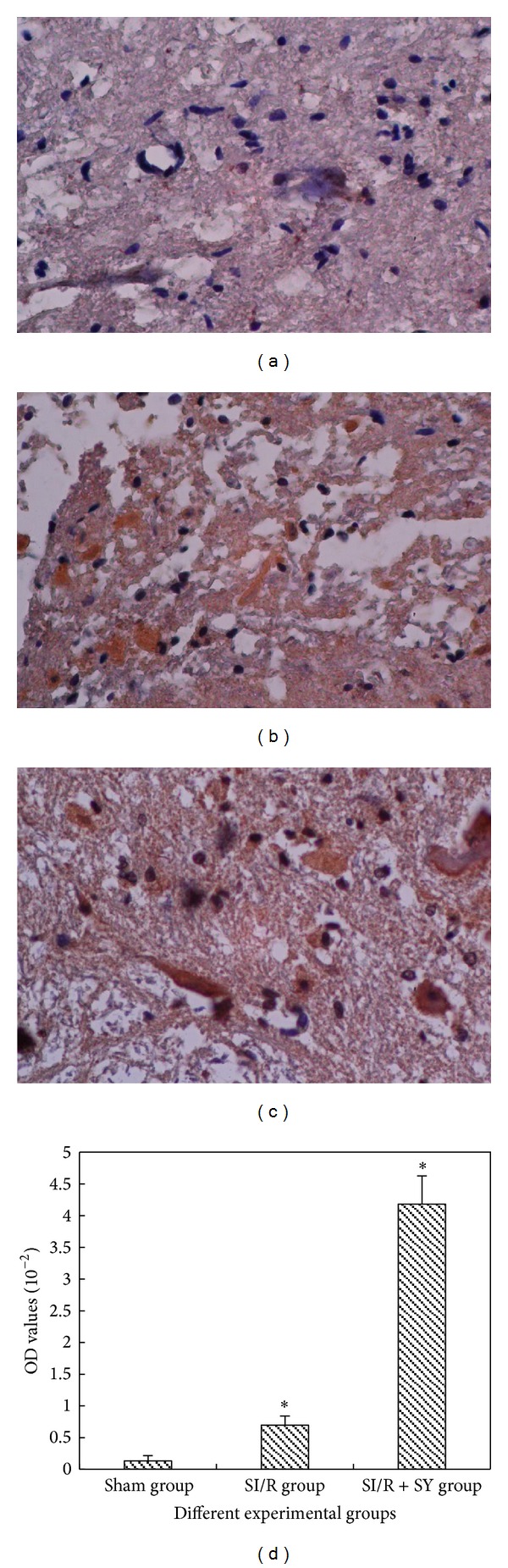
((a)–(c)) Bcl-2 protein expression in the spinal cord anterior horn at the same magnification of 200 times, and OD values of Bcl-2 protein staining were measured and analyzed in image (d); ∗ indicate comparison with the two other groups, *P* < 0.05.

*LM Observation of Bax and Bcl-2 Protein Expression.* Spinal cord sections were, respectively, stained with Bax and Bcl-2 and were observed at the high LM level (200 times magnification, Figures 8 and 9). The results showed that Bax protein was more intensely expressed in the SI/R group than in the other groups (*P* < 0.05, Figure 8(d)), but Bcl-2 expression of SI/R + SY group was obviously intense (Figures 9(a)–9(c)), and its OD value was also higher than that of the Control group and SI/R group (*P* < 0.05, Figure 9(d)).

## 4. Discussion

Spinal cord blood supply is significantly segmental, and its collateral circulation is relatively poor, so that it is easy to suffer from ischemia damage [5]. Due to constant and seldom variation of vascular distribution in the lumbar region, the degree of spinal cord injury is stable with higher repeatability and less complications. Therefore, we constructed spinal cord ischemia reperfusion model in rabbits as Zivin's description [5].

The cell membranes of spinal cord neurons are rich in lipid content and large amounts of catecholamines and unsaturated fatty acids, so that they are more susceptible to oxygen free radical attack [9]. Reactive oxygen species-mediated lipid peroxidation plays an important role in ischemia reperfusion injuries in various organs [10–12]. After spinal cord suffered the injury, the lipid peroxidation reaction was extensively activated and MDA was largely produced, but SOD activities markedly decreased. Increasing SOD activity has been shown to evidently attenuate spinal cord injury [2].

Many traditional Chinese medicines are used as the natural oxygen free radical scavenger and have the advantage of lowering toxic adverse actions of the nonnatural products, which have protective effects against ischemia reperfusion injury [13]. Safflower and its extracts play an important role of inhibiting lipid peroxidation and clearing oxygen free radical. Safflower injection can significantly increase the activities of glutathione peroxidase (SE-GSHPX) and SOD and decrease MDA contents in ischemia reperfusion injury myocardium. The present study showed that increase in MDA and decrease in SOD were part of the reaction of the entire body in response to SCIRI, and they were closely correlative with the degree of injury. Immediately after reperfusion (reperfusion 0 hour), the lipid peroxidation of spinal cord neurons in the reperfusion groups (including SY treatment group) began to intensify as observed from changes in MDA contents, and at 4 hours after reperfusion the plasma level of MDA production in the SI/R group was further increased and SOD activity was further decreased. These results indicated that lipid peroxidation had been triggered in the ischemia stage, and then in the reperfusion stage it further increased and aggravated ischemia reperfusion injury in the spinal cord. However, lipid peroxidation in the SI/R + SY group was attenuated (which means lower MDA content and higher SOD activity) at the different time points after reperfusion. These results indicated that SY played an important role in the spinal cord protection by relieving lipid peroxidation.

The study has confirmed that IL-8 is an important type of cytokine, which takes part in inflammation reaction [14]. It primarily plays a role through the cytomembrane surface receptors. Compared with the other cells, neutrophil granulocyte surfaces significantly raise expressed IL-8 receptors, so that IL-8 mainly induces chemotaxis of neutrophil chemotactic and may be an important chemotactic factor in mediating neutrophil aggregation [15]. The present results showed that, compared with sham-cont group, IL-8 contents in the reperfusion group without SY treatment obviously increased. Moreover, with the progression of reperfusion, IL-8 level gradually increased and reached the peak after reperfusion 12 h before it started to decrease. In the SI/R + SY group its change had the same tendency, but it was lower than that in the SI/R group at various time points. This result indicated that SY might inhibit IL-8 expression and inflammatory reaction induced by SCIRI.

The mechanism of cell apoptosis is very complicated and is influenced by many factors, especially by gene regulation. caspase family, a type of calcium-dependent Cysteine protease, is the key protease triggering cell apoptosis and is present in the whole process of apoptosis [16]. The current study confirmed that the changes of caspase-3 expression were in line with the changes in cell apoptosis after SCIRI, suggesting that caspase-3 could be used as biochemical index in assessing post-ischemic spinal cord injury. The present result showed that the expression level of caspase-3 in SI/R + SY group was obviously lower than that in SI/R group, which suggests that inhibiting caspase-3 expression may represent a mechanism by which SY confers protection against SCIRI. The process of apoptosis is regulated by a complex interaction of proapoptotic (Bax group of proteins) and antiapoptotic (Bcl-2 family of proteins) mitochondrial membrane proteins and the activation of effector caspase [17]. The findings from studying the neurons in animal models of spinal cord injury should help to enhance our understanding of the morphologic features of damaged neurons, to identify the mechanism of neuronal damage, and, therefore, to develop more effective therapies against SCIRI.

## 5. Conclusions

SI/R remarkably affected the neurological function of hind limbs and activated the lipid peroxidation reaction and promoted the inflammatory cytokine release. SY can reduce postischemic lipid peroxidation and inflammatory response and effectively attenuate SCIRI in rabbits.

## Figures and Tables

**Table 1:** Basic vital signs of all animals at the different time points.

Item	Cont. group	SI/R group	SI/R + SY group
HR (order/rate)			
*T*0	213 ± 8.5	218 ± 10.6	224 ± 7.5
*T*1	213 ± 17.4	207 ± 15.2	216 ± 12.3
*T*2	215 ± 9.1	212 ± 14.8	205 ± 10.0
*T*3	204 ± 15.0	213 ± 7.9	213 ± 14.6
*T*4	213 ± 11.9	215 ± 16.7	213 ± 9.7
MAP (mmHg)			
*T*0	90 ± 7.1	93 ± 7.6	92 ± 4.9
*T*1	89 ± 5.8	88 ± 5.4	91 ± 5.6
*T*2	91 ± 6.5	87 ± 6.2	88 ± 8.1
*T*3	88 ± 6.2	90 ± 4.3	90 ± 4.9
*T*4	95 ± 3.7	91 ± 4.8	93 ± 7.3

HR: heart rate; MAP: mean arterial pressure; *T*1: 4 h after reperfusion; *T*2: 12 h after reperfusion; *T*3: 24 h after reperfusion; *T*4: 48 h after reperfusion.
